# Supplementary material for: A yeast phenomic model for the influence of Warburg metabolism on genetic buffering of doxorubicin
Source: Cancer Metab. 2019 Oct 23;7:9. doi: 10.1186/s40170-019-0201-3 (PMC6806529; doi:10.1186/s40170-019-0201-3)

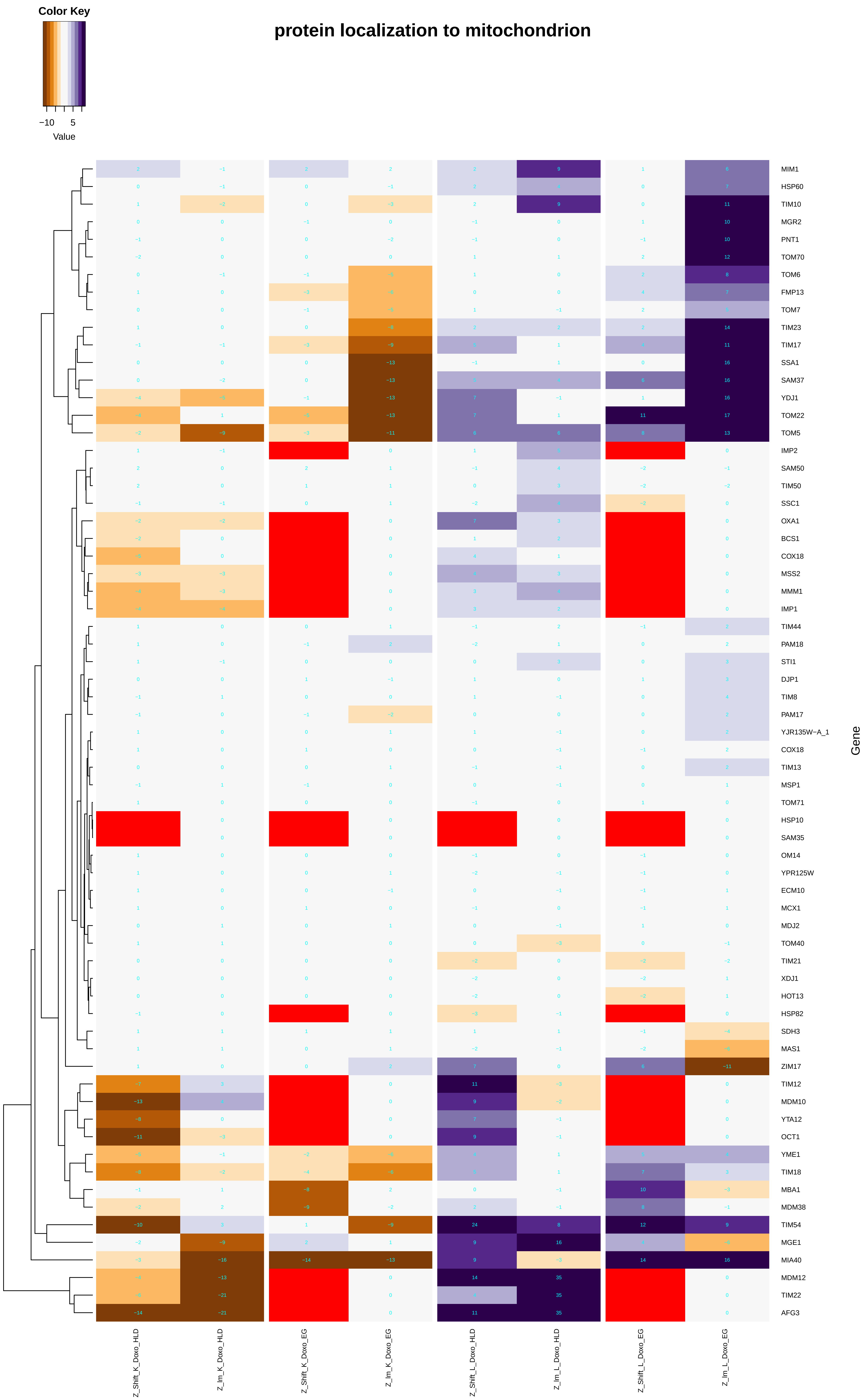
Color Key
−10
5
Value
protein localization to mitochondrion
MIM1
HSP60
TIM10
MGR2
PNT1
TOM70
TOM6
FMP13
TOM7
TIM23
TIM17
SSA1
SAM37
YDJ1
TOM22
TOM5
IMP2
SAM50
TIM50
SSC1
OXA1
BCS1
COX18
MSS2
MMM1
IMP1
TIM44
PAM18
STI1
DJP1
TIM8
PAM17
YJR135W−A_1
COX18
TIM13
MSP1
TOM71
HSP10
SAM35
OM14
YPR125W
ECM10
MCX1
MDJ2
TOM40
TIM21
XDJ1
HOT13
HSP82
SDH3
MAS1
ZIM17
TIM12
MDM10
YTA12
OCT1
YME1
TIM18
MBA1
MDM38
TIM54
MGE1
MIA40
MDM12
TIM22
AFG3
Gene
Z_Shift_K_Doxo_HLD
Z_Im_K_Doxo_HLD
Z_Shift_K_Doxo_EG
Z_Im_K_Doxo_EG
Z_Shift_L_Doxo_HLD
Z_Im_L_Doxo_HLD
Z_Shift_L_Doxo_EG
Z_Im_L_Doxo_EG

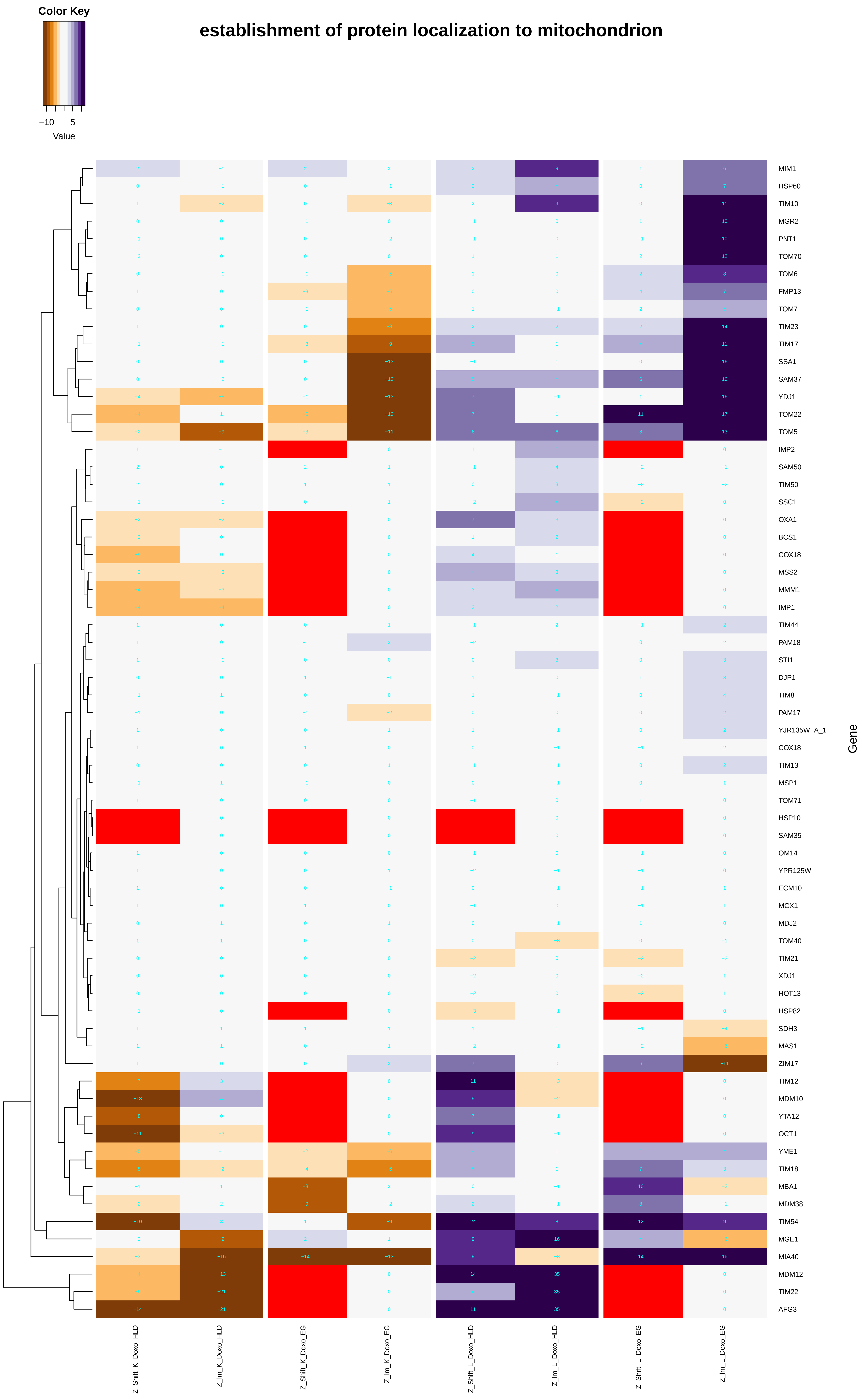
Color Key
−10
5
Value
establishment of protein localization to mitochondrion
MIM1
HSP60
TIM10
MGR2
PNT1
TOM70
TOM6
FMP13
TOM7
TIM23
TIM17
SSA1
SAM37
YDJ1
TOM22
TOM5
IMP2
SAM50
TIM50
SSC1
OXA1
BCS1
COX18
MSS2
MMM1
IMP1
TIM44
PAM18
STI1
DJP1
TIM8
PAM17
YJR135W−A_1
COX18
TIM13
MSP1
TOM71
HSP10
SAM35
OM14
YPR125W
ECM10
MCX1
MDJ2
TOM40
TIM21
XDJ1
HOT13
HSP82
SDH3
MAS1
ZIM17
TIM12
MDM10
YTA12
OCT1
YME1
TIM18
MBA1
MDM38
TIM54
MGE1
MIA40
MDM12
TIM22
AFG3
Gene
Z_Shift_K_Doxo_HLD
Z_Im_K_Doxo_HLD
Z_Shift_K_Doxo_EG
Z_Im_K_Doxo_EG
Z_Shift_L_Doxo_HLD
Z_Im_L_Doxo_HLD
Z_Shift_L_Doxo_EG
Z_Im_L_Doxo_EG

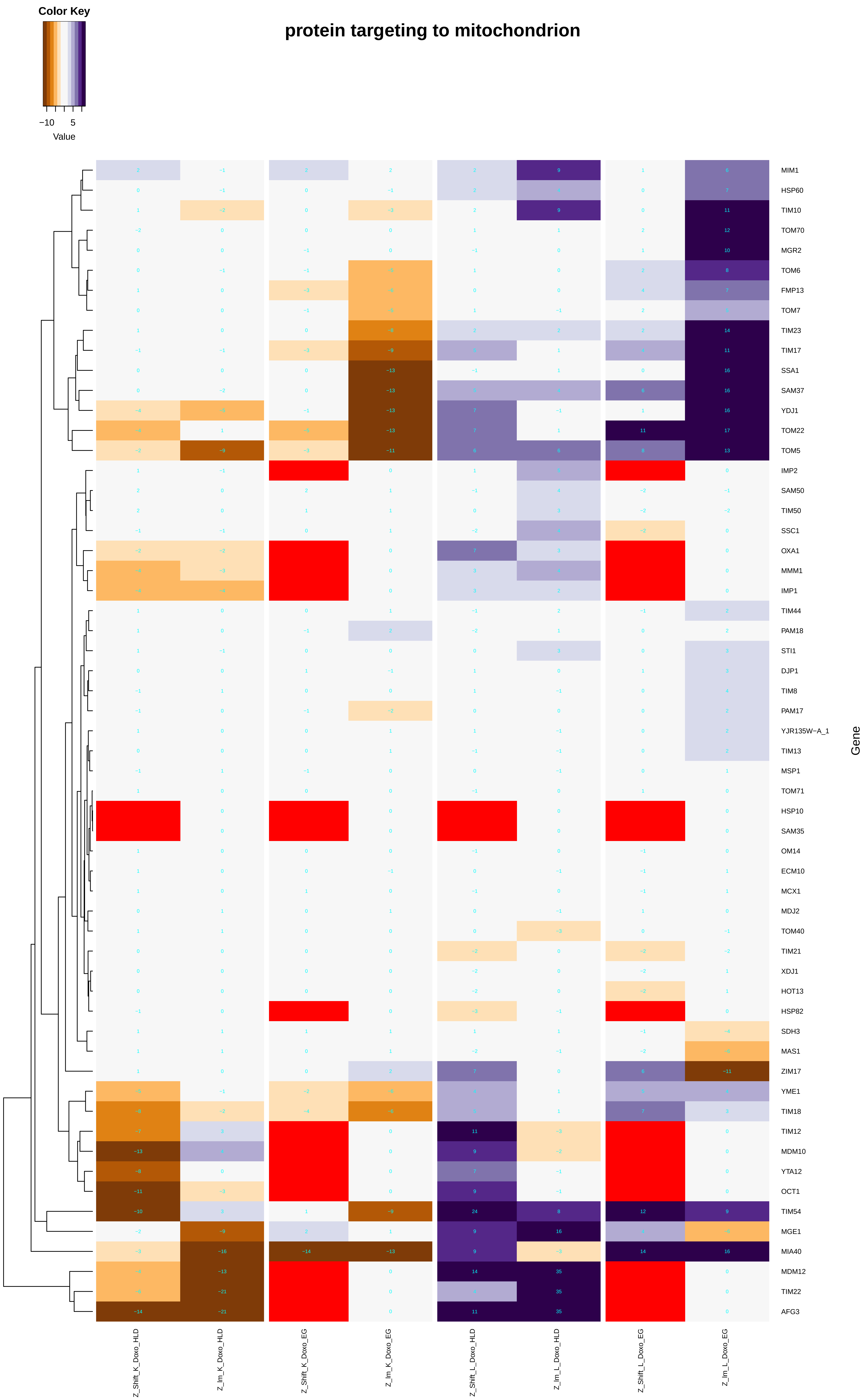

Color Key
−10
5
Value
protein targeting to mitochondrion
MIM1
HSP60
TIM10
TOM70
MGR2
TOM6
FMP13
TOM7
TIM23
TIM17
SSA1
SAM37
YDJ1
TOM22
TOM5
IMP2
SAM50
TIM50
SSC1
OXA1
MMM1
IMP1
TIM44
PAM18
STI1
DJP1
TIM8
PAM17
YJR135W−A_1
TIM13
MSP1
TOM71
HSP10
SAM35
OM14
ECM10
MCX1
MDJ2
TOM40
TIM21
XDJ1
HOT13
HSP82
SDH3
MAS1
ZIM17
YME1
TIM18
TIM12
MDM10
YTA12
OCT1
TIM54
MGE1
MIA40
MDM12
TIM22
AFG3
Gene
Z_Shift_K_Doxo_HLD
Z_Im_K_Doxo_HLD
Z_Shift_K_Doxo_EG
Z_Im_K_Doxo_EG
Z_Shift_L_Doxo_HLD
Z_Im_L_Doxo_HLD
Z_Shift_L_Doxo_EG
Z_Im_L_Doxo_EG

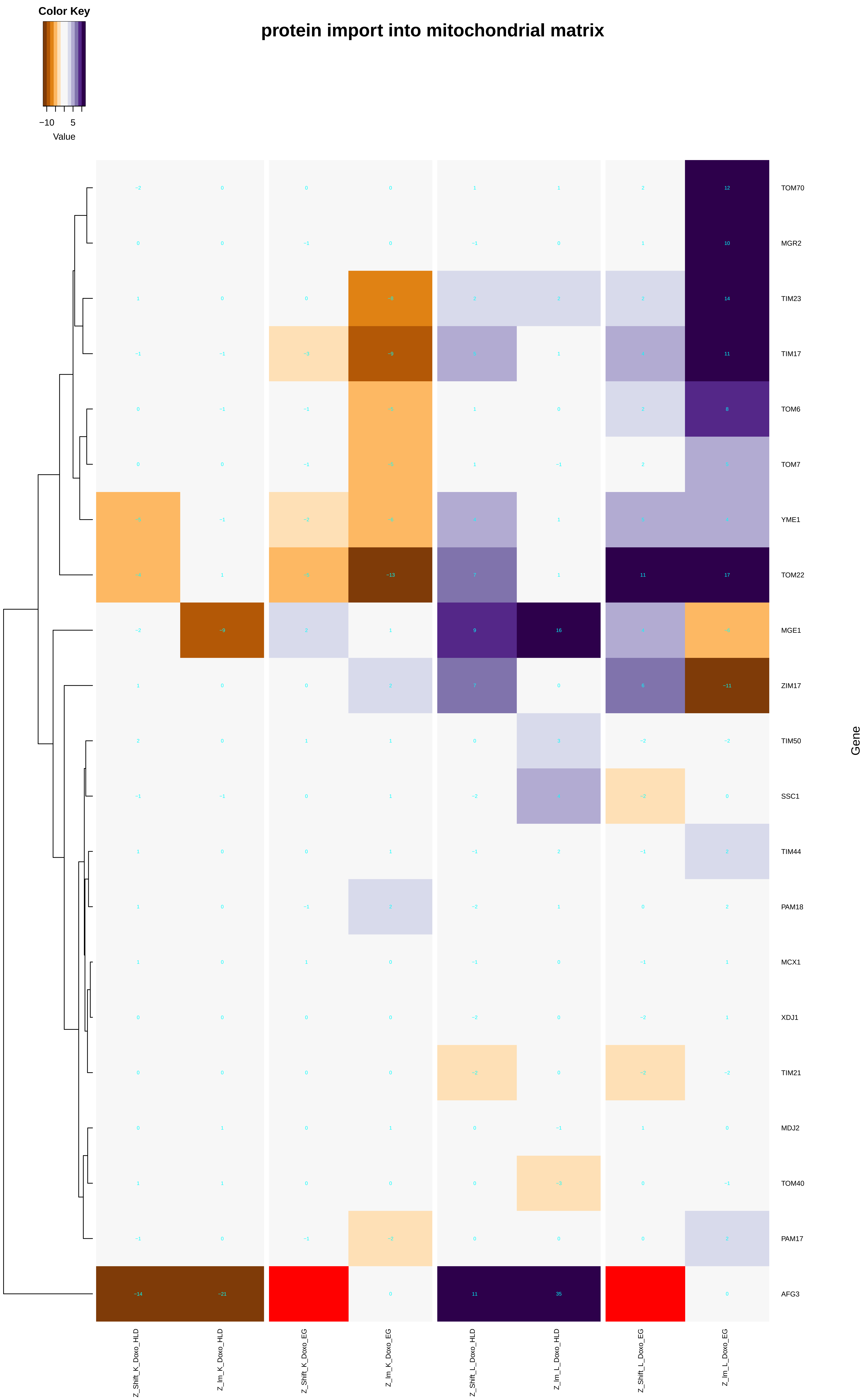

protein import into mitochondrial matrix
Color Key
−10
5
Value
TOM70
MGR2
TIM23
TIM17
TOM6
TOM7
YME1
TOM22
MGE1
ZIM17
TIM50
SSC1
TIM44
PAM18
MCX1
XDJ1
TIM21
MDJ2
TOM40
PAM17
AFG3
Gene
Z_Shift_K_Doxo_HLD
Z_Im_K_Doxo_HLD
Z_Shift_K_Doxo_EG
Z_Im_K_Doxo_EG
Z_Shift_L_Doxo_HLD
Z_Im_L_Doxo_HLD
Z_Shift_L_Doxo_EG
Z_Im_L_Doxo_EG

protein import into mitochondrial intermembrane space

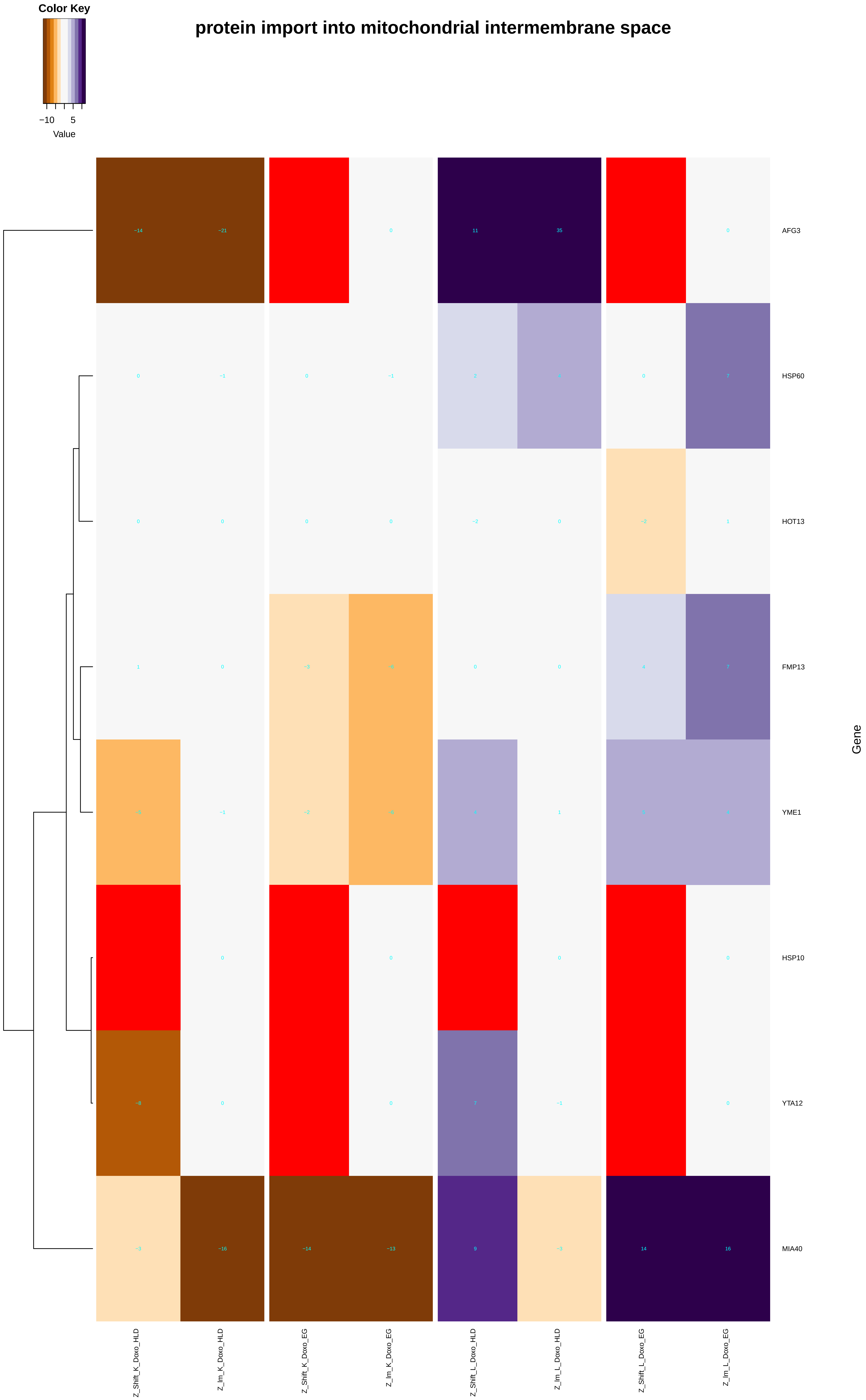

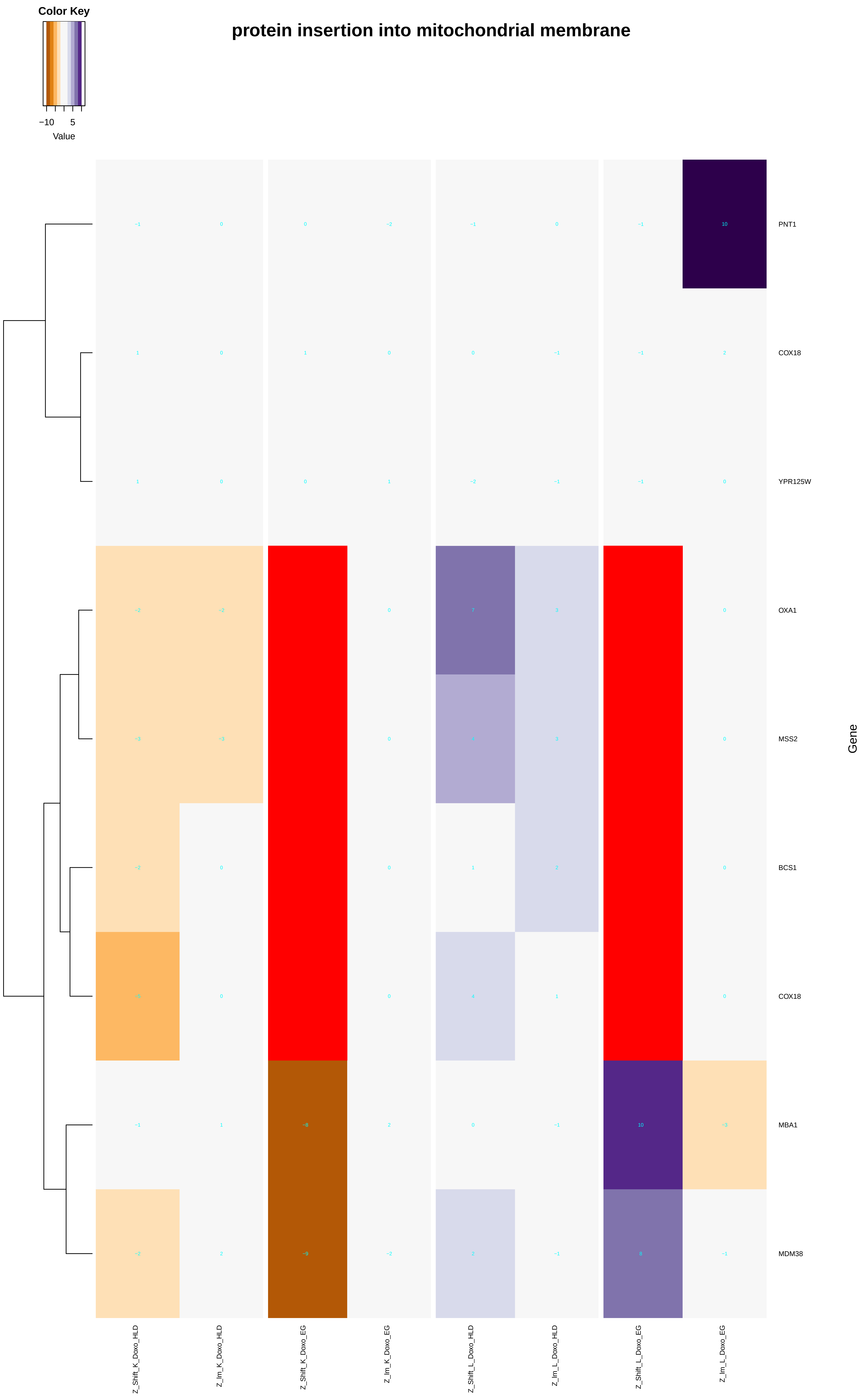
Color Key
−10
5
Value
protein insertion into mitochondrial membrane
PNT1
COX18
YPR125W
OXA1
MSS2
BCS1
COX18
MBA1
MDM38
Gene
Z_Shift_K_Doxo_HLD
Z_Im_K_Doxo_HLD
Z_Shift_K_Doxo_EG
Z_Im_K_Doxo_EG
Z_Shift_L_Doxo_HLD
Z_Im_L_Doxo_HLD
Z_Shift_L_Doxo_EG
Z_Im_L_Doxo_EG

establishment of protein localization to mitochondrial membrane

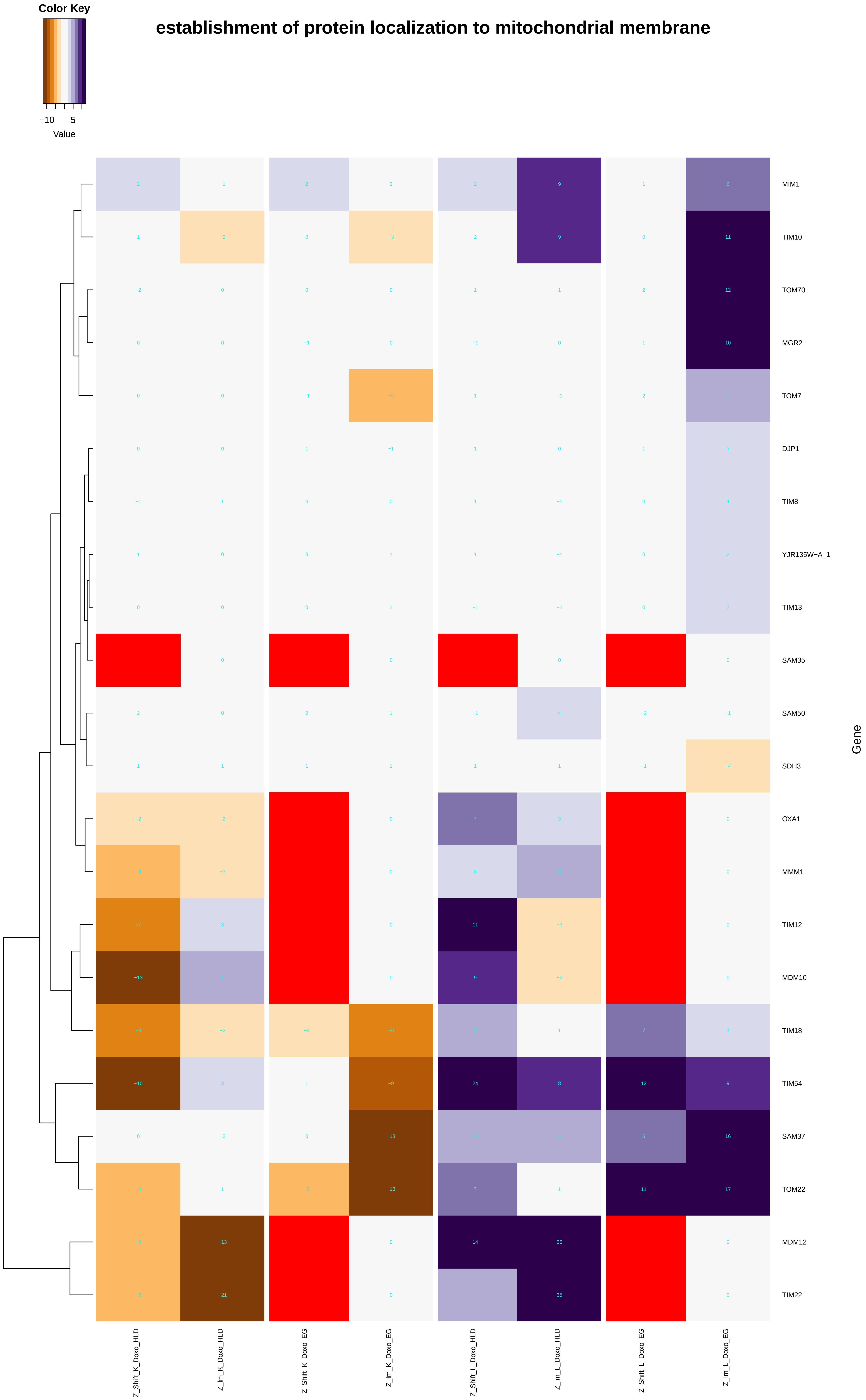

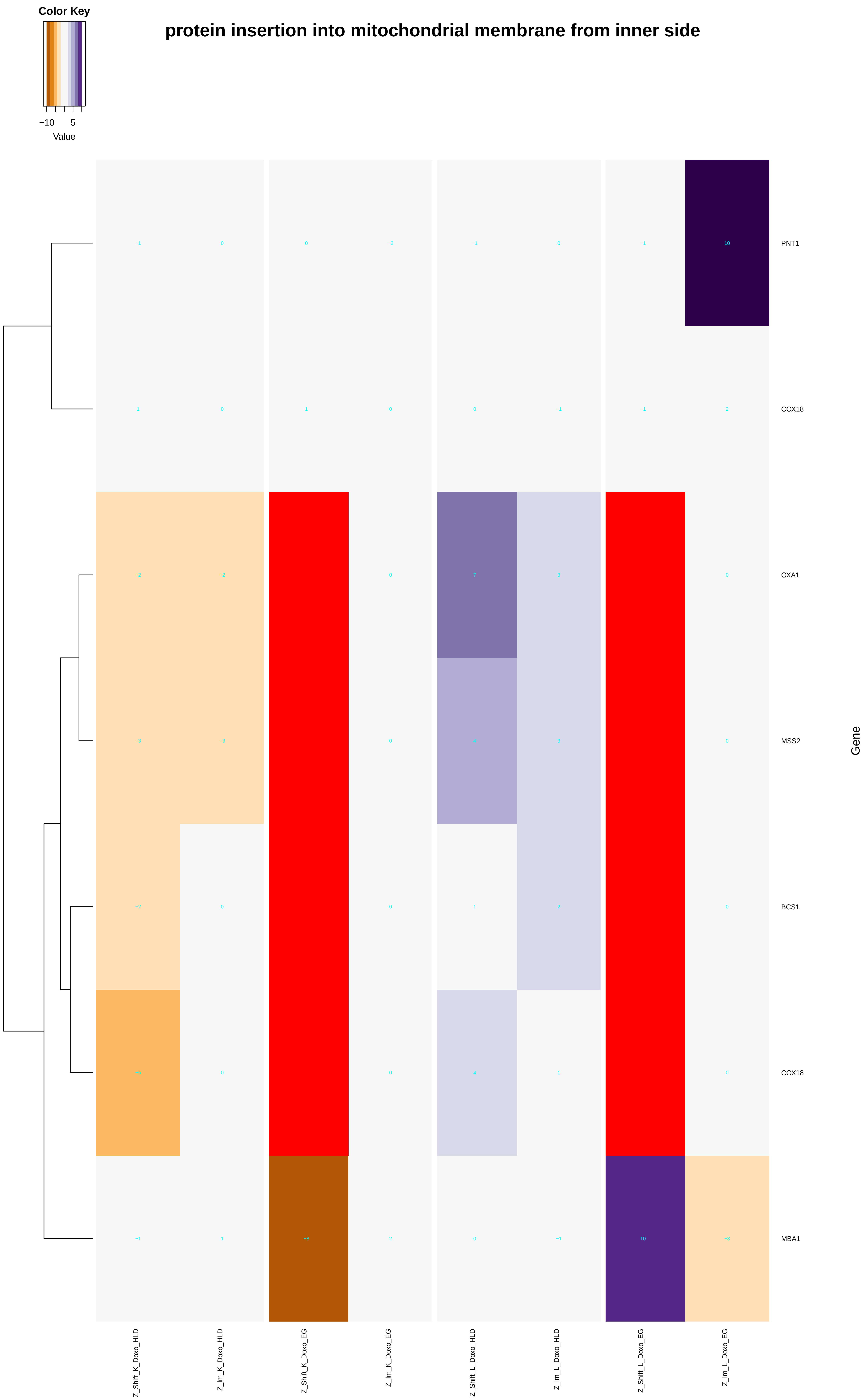

Color Key
−10
5
Value
protein insertion into mitochondrial membrane from inner side
PNT1
COX18
OXA1
MSS2
BCS1
COX18
MBA1
Gene
Z_Shift_K_Doxo_HLD
Z_Im_K_Doxo_HLD
Z_Shift_K_Doxo_EG
Z_Im_K_Doxo_EG
Z_Shift_L_Doxo_HLD
Z_Im_L_Doxo_HLD
Z_Shift_L_Doxo_EG
Z_Im_L_Doxo_EG

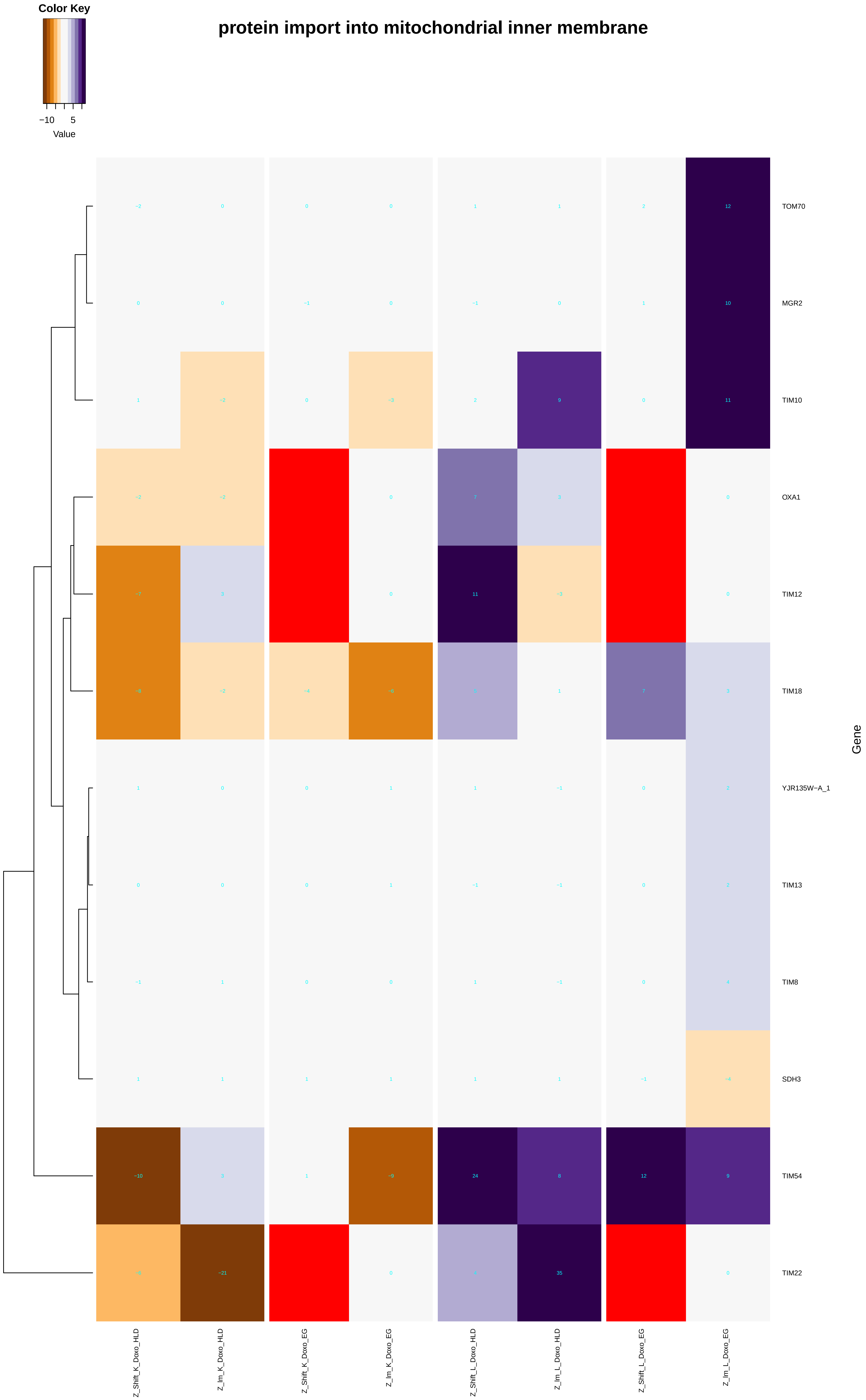
Color Key
−10
5
Value
protein import into mitochondrial inner membrane
TOM70
MGR2
TIM10
OXA1
TIM12
TIM18
YJR135W−A_1
TIM13
TIM8
SDH3
TIM54
TIM22
Gene
Z_Shift_K_Doxo_HLD
Z_Im_K_Doxo_HLD
Z_Shift_K_Doxo_EG
Z_Im_K_Doxo_EG
Z_Shift_L_Doxo_HLD
Z_Im_L_Doxo_HLD
Z_Shift_L_Doxo_EG
Z_Im_L_Doxo_EG

protein import into mitochondrial outer membrane

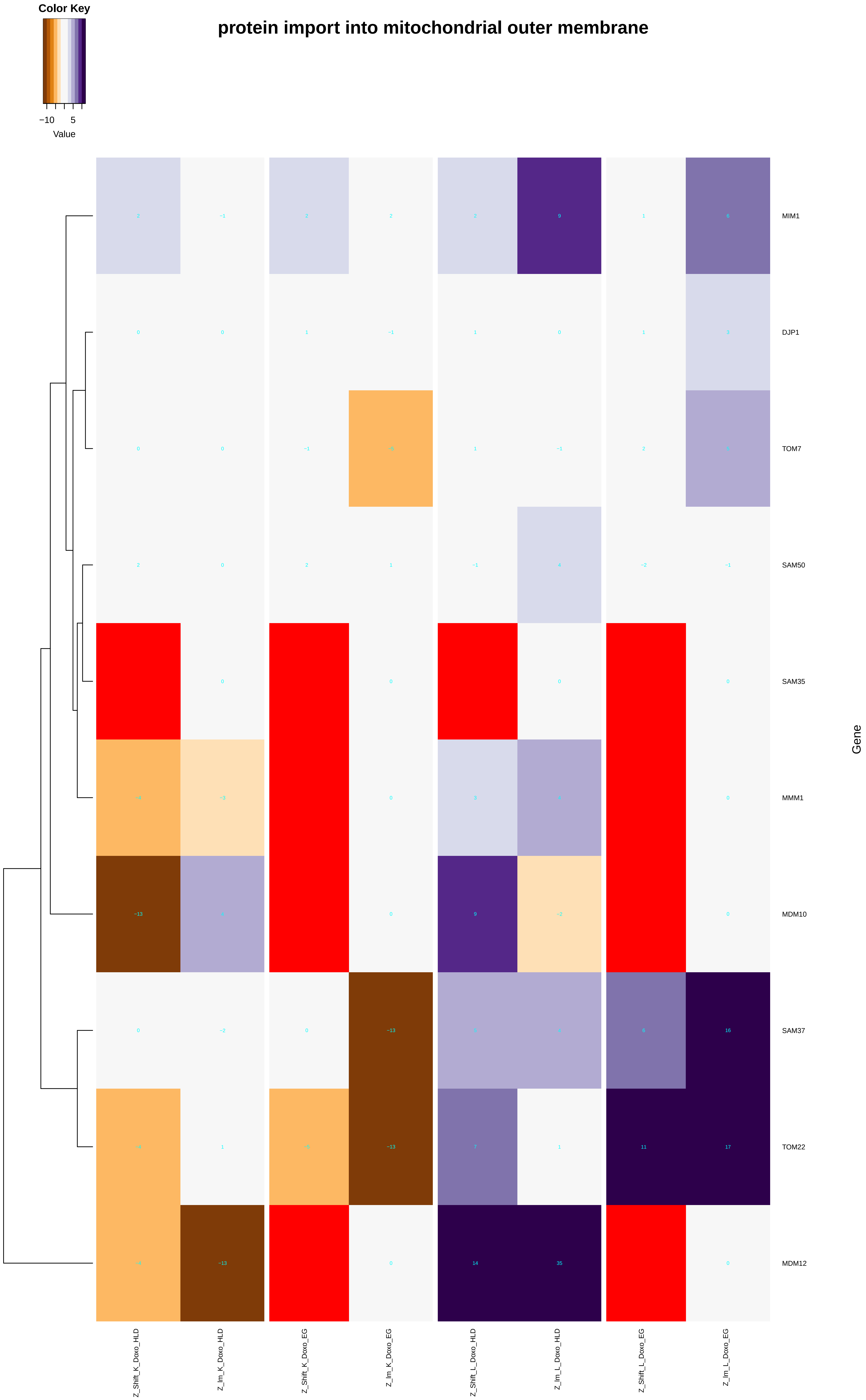

Supplement: Supplementary file 9 — Additional file 9. GO term-specific heatmaps for REMc/GTF-enriched clusters. GO term-specific heatmaps for significant GO process terms were generated as described in methods and Figs. 3 and 4. Any related child terms are presented in subsequent pages of the parent file name. GO terms with more than 100 children, with 2 or fewer genes annotated to the term, or a file size over 300KB are not shown. All heatmaps are generated with the same layout (see Figs. 3 and 4). [file 40170_2019_201_MOESM9_ESM.bz2 › Additional_File9_GOTermHeatmaps/Additional_File9_GOTermHeatmap/protein_localization_to_mitochondrion.pdf]
